# Differential levels of plasma biomarkers of neurodegeneration in Lewy body dementia, Alzheimer’s disease, frontotemporal dementia and progressive supranuclear palsy

**DOI:** 10.1136/jnnp-2021-327788

**Published:** 2022-01-25

**Authors:** Leonidas Chouliaras, Alan Thomas, Maura Malpetti, Paul Donaghy, Joseph Kane, Elijah Mak, George Savulich, Maria A Prats-Sedano, Amanda J Heslegrave, Henrik Zetterberg, Li Su, James Benedict Rowe, John Tiernan O’Brien

**Affiliations:** 1 Department of Psychiatry, University of Cambridge School of Clinical Medicine, Cambridge, UK; 2 Translational and Clinical Research Institute, Newcastle University, Newcastle, UK; 3 Department of Clinical Neurosciences, University of Cambridge School of Clinical Medicine, Cambridge, UK; 4 Centre for Public Health, Queen’s University Belfast, Belfast, UK; 5 Department of Neurodegenerative Disease, UCL Queen Square Institute of Neurology, London, UK; 6 Dementia Research Insitute, UCL, London, UK; 7 Department of Psychiatry and Neurochemistry, Institute of Neuroscience and Physiology, The Sahlgrenska Academy, University of Gothenburg, Gothenburg, Sweden; 8 Clinical Neurochemistry Laboratory, Sahlgrenska University Hospital, Mölndal, Sweden; 9 Hong Kong Center for Neurodegenerative Diseases, Hong Kong, China; 10 Department of Neuroscience, University of Sheffield, Sheffield, UK; 11 Cambridge University Hospitals NHS Foundation Trust, Cambridge, UK

## Abstract

**Objectives:**

This longitudinal study compared emerging plasma biomarkers for neurodegenerative disease between controls, patients with Alzheimer’s disease (AD), Lewy body dementia (LBD), frontotemporal dementia (FTD) and progressive supranuclear palsy (PSP).

**Methods:**

Plasma phosphorylated tau at threonine-181 (p-tau181), amyloid beta (Aβ)42, Aβ40, neurofilament light (NfL) and glial fibrillar acidic protein (GFAP) were measured using highly sensitive single molecule immunoassays (Simoa) in a multicentre cohort of 300 participants (controls=73, amyloid positive mild cognitive impairment (MCI+) and AD dementia=63, LBD=117, FTD=28, PSP=19). LBD participants had known positron emission tomography (PET)-Aβ status.

**Results:**

P-tau181 was elevated in MCI+AD compared with all other groups. Aβ42/40 was lower in MCI+AD compared with controls and FTD. NfL was elevated in all dementias compared with controls while GFAP was elevated in MCI+AD and LBD. Plasma biomarkers could classify between MCI+AD and controls, FTD and PSP with high accuracy but showed limited ability in differentiating MCI+AD from LBD. No differences were detected in the levels of plasma biomarkers when comparing PET-Aβ positive and negative LBD. P-tau181, NfL and GFAP were associated with baseline and longitudinal cognitive decline in a disease specific pattern.

**Conclusion:**

This large study shows the role of plasma biomarkers in differentiating patients with different dementias, and at monitoring longitudinal change. We confirm that p-tau181 is elevated in MCI+AD, versus controls, FTD and PSP, but is less accurate in the classification between MCI+AD and LBD or detecting amyloid brain pathology in LBD. NfL was elevated in all dementia groups, while GFAP was elevated in MCI+AD and LBD.

## Introduction

Clinical diagnostics and clinical trials will benefit from scalable non-invasive biomarkers moving beyond positron emission tomography (PET) imaging and cerebrospinal fluid (CSF) analysis to blood based assays.^1^ Recent years have seen the emergence of many new plasma biomarkers, but head-to-head comparisons, across multiple dementias and over time, are required to assess their potential for differential diagnosis and trials monitoring.^2^ Here we jointly evaluate a set of biomarkers in Alzheimer’s disease (AD; the most common type of neurodegenerative dementia), Lewy body dementia (LBD; dementia with Lewy bodies (DLB) and Parkinson’s disease dementia (PDD)), frontotemporal dementia (FTD) and progressive supranuclear palsy (PSP).^3^ Biomarkers are most advanced and validated for AD, including preclinical stages, for diagnosis and in trials of disease-modifying treatments.^3 4^ For example, biomarkers for AD may use the ‘A/T/N’ classification system referring to levels of amyloid beta (Aβ; A), tau (T) and neurodegeneration/neuronal injury (N). Amyloid is measured using either CSF levels of the 42 and 40 amino acid form of Aβ or using PET with ligands binding to Aβ (PET-Aβ). Tau is measured using CSF levels of phosphorylated tau or PET with ligands binding to tau and neurodegeneration is measured using MRI, fluorodeoxyglucose-PET or CSF total tau.^5 6^ However, such biomarkers are challenging to apply at scale, or frequently, and also need evidence of their performance and differences across multiple dementias.

Technological advances now allow measurement of such multiple biomarkers in blood plasma with great potential for the development of blood biomarkers for diagnosis and tracking of AD and other neurodegenerative diseases.^7^ Plasma levels of phosphorylated tau at threonine 181 (p-tau181) at threonine 217 (p-tau217) and at threonine-231 (p-tau231) are promising blood biomarkers for detecting AD pathology.^1 4–8^ Studies so far have shown high levels of correlation between p-tau181 and p-tau217 with their CSF counterparts and with PET tau.^6 9^ They are markedly elevated in blood samples of patients with AD when compared with controls and other neurodegenerative conditions, have high accuracy in discriminating between AD and FTD and correlate well with postmortem burden of Aβ and tau neuropathology,^2 4–6 9–11^ though their ability to distinguish AD from LBD is less clear and only a small number of LBD cases have been included in published studies comparing these biomarkers. They also have a prognostic value to detect preclinical AD and predict both longitudinal decline and conversion from mild cognitive impairment (MCI) to AD.^5 7 12–14^ In parallel, measurement of the ratio of Aβ42 and Aβ40 in blood using highly sensitive assays is a good proxy for presence of amyloid brain pathology and diagnosis of AD but may be confounded by peripheral amyloid production.^15 16^ Neurofilament light (NfL), a marker of neuroaxonal damage, in CSF and plasma has been established as a biomarker of neurodegeneration across different types of dementia.^17 18^ In parallel, neuroinflammation has been linked with the development of dementia and plasma glial fibrillar acidic protein (GFAP), a marker of astrocytic activation, is elevated early in AD and in cognitively normal older adults who are at risk of AD based on brain Aβ load.^19 20^ Moreover, plasma GFAP was associated with AD pathology in MCI and predicted conversion to AD.^21^

Less is known about the use of these plasma biomarkers in LBD, whether they can improve the diagnosis of LBD or distinguish the common AD-copathology in LBD.^22^ LBD is driven by cortical Lewy body pathology but approximately half of patients with LBD have coexisting AD amyloid pathology.^23^ P-tau181 and p-tau217 may be able to identify LBD cases with coexisting AD pathology as measured with PET tau and CSF Aβ.^24^ People with non-AD pathologies, including Lewy bodies, show progressively elevated p-tau181 with ageing up until death, while those with AD show increases in plasma p-tau181 very early on in the disease course.^4^ Evidence is required for the ability of plasma biomarkers for neurodegeneration to distinguish between those with clinical diagnoses of AD, LBD and copathology.

The present study had four primary aims. First, to measure the levels of the plasma biomarkers of neurodegeneration (p-tau181, Aβ42/40 ratio, NfL and GFAP) across a multicentre memory clinical cohort of older participants with PET-Aβ-positive MCI (MCI+) and AD (MCI+AD), LBD, FTD, PSP and controls. Second, to test the ability of these biomarkers to accurately classify a diagnosis of MCI+AD compared with controls, LBD, FTD and PSP. Third to test the ability of plasma biomarkers to detect the presence of AD pathology in LBD and last to examine the relation between plasma biomarkers and baseline cognitive function and subsequent longitudinal decline. We predicted that plasma biomarkers would be differentially elevated among the different types of dementia and that p-tau181 and Aβ42/40 would distinguish AD cases from the different forms of dementia while detecting coexistent AD pathology in LBD.

## Materials and Methods

### Participant characteristics

The study included 300 participants from five diagnostic groups (see table 1 for summary characteristics). Participants were above the age of 50. Exclusion criteria included an acute infection, major concurrent psychiatric illness; severe physical illness; a history of other significant neurological illness. Participants with capacity gave their written informed consent to take part in the study. For those who lacked capacity, their participation followed the consultee process in accordance with the UK law.

One hundred and forty-five participants were included from the Neuroimaging of Inflammation in Memory and Related Other Disorders (NIMROD); 76 participants from the Amyloid Imaging for Phenotyping Lewy Body Dementia (AMPLE), 29 from the Clinical Biomarkers for Dementia Research, 27 from the ^123^I-MIBG in Dementia with Lewy bodies as A marker for Sympathetic denervation (MIDAS) and 23 from the Multimodal Imaging of Lewy Body Disorders study (MILOS). Detailed background, clinical diagnostic criteria, clinical and neuroimaging findings relating to these cohorts have been published previously.^23 25–27^ The pooled study cohort comprised of a total of 73 non-demented controls, 63 participants in the AD spectrum consisting of 14 PET-Aβ-positive MCI (MCI+) and 49 AD dementia participants who were combined and analysed as a single MCI+AD group as in our previous studies, reflecting different stages on the AD spectrum, 117 LBD (110 DLB and 7 PDD, 59 with PET-Aβ status), 28 patients with FTD and 19 patients with PSP. The FTD group included 10 patients with behavioural variant FTD (bvFTD), 9 patients with non-fluent primary progressive aphasia (nfPPA) and 7 patients with semantic variant PPA (svPPA). Three patients with bvFTD were found to carry the C9orf72 mutation, two bvFTD carried the MAPT mutation, one bvFTD and one nfPPA carried GRN mutation. All FTD subtypes were combined to a single group due to the low number of samples available. Participants were recruited from specialist memory clinics in and around Cambridgeshire and the North of England, the Dementia and Neurodegeneration specialty of the UK Clinical Research Network, the Join Dementia Research platform (www.joindementiaresearch.nihr.ac.uk) as well as from cognitively healthy spouses and partners of participants.

Healthy controls had mini-mental state examination >26 with no acute physical illness, no cognitive complaints and who were independent in function and instrumental activities of daily living. MCI PET-Aβ-positive participants were defined by presence of subjective memory reports, no impairment in activities of daily living and amyloid positivity using Pittsburgh Compound B (PIB) PET using a cutpoint of 19 in the centiloid scale.^28^ AD participants fulfilled the criteria for AD dementia defined as per the National Institute on Aging-Alzheimer’s Association Criteria.^29^ Probable DLB was defined by both the 2005 and 2017 consensus criteria and PDD was defined by the Movements Disorders Society clinical diagnosis criteria for PDD.^30 31^ DLB and PDD were combined in a group representing the LBD group. FTD diagnoses were based on the criteria defined clinically by Rascovsky *et al* for behavioural variant of FTD and Gorno-Tempini *et al*^32 33^ for PPA variants. Participants with PSP were recruited initially according to the Litvan *et al*^34^ criteria (modified by a relaxation of the falls criterion to falls within 3 years as suggested by the NNIPPS-PSP study group,^34^ and later reclassified according to the MDS-PSP 2017 criteria for PSP-Richardson’s syndrome.^35^

### Clinical assessments

Participants underwent clinical examination with cognitive and neuropsychiatric assessment at baseline. These were repeated annually for up to 3 years for the NIMROD and MILOS cohorts and at 1 year for the AMPLE and MIDAS cohorts. Cognitive function in this study was measured using the Addenbrooke’s Cognitive Examination Revised version (ACE-R). The ACE-R incorporates five domains of cognitive function (attention/orientation, memory, verbal fluency, language, visuospatial) hand has been shown to capture deficits observed in the neuro-degenerative dementias included in this cohort.^27 36 37^ Longitudinal ACE-R data were available for a total of 185 out of 300 participants (control=52, MCI+AD=50, LBD=52, FTD=15, PSP=16). Nine of the MCI-PET-Aβ-positive participants were clinically diagnosed as converting to AD at subsequent follow-up visits.

### PET-Aβ in LBD

Imaging was performed at baseline. Details of the MRI and PET acquisition and analysis have been published previously.^23 25^ In summary, PET-Aβ imaging data were available for 59 participants with LBD. For the NIMROD and MILOS cohort 550 MBq of [11^C^] PIB PET imaging was carried out using a GE Advance PET scanner (GE Healthcare) or a GE Discovery 690 PET/CT, with attenuation correction provided by a transmission scan or a low dose CT scan, with 550 MBq of PIB injected as a bolus and imaging performed for 30 min starting at 40 min post injection. Participants were considered PET-Aβ-positive using a cutpoint of 19 on the centiloid scale.^28^

For the AMPLE cohort imaging was performed using a Siemens Biograph-40 PET-CT scanner. Participants were given a 370 MBq intravenous injection of ^18^F-florbetapir (Amyvid). PET imaging was carried out for 15 min, commencing 30−50 min after injection. Attenuation correction was performed using CT scan data. Amyloid PET images were visually rated as positive or negative based on the manufacturer’s criteria by a panel of five raters.^23^

### Sample collection and processing

Blood samples were obtained by venepuncture and collected in EDTA tubes. They were centrifuged to isolate plasma, aliquoted and stored at −70°C until further analyses. Plasma assays were conducted at the UK Dementia Research Institute biomarker laboratory. Plasma samples were thawed on wet ice, centrifuged at 500× g for 5 min at 4°C. Calibrators (neat) and samples (plasma: 1:4 dilution) were measured in duplicates. The plasma assays measured were the Quanterix Simoa Human Neurology 4-Plex E assay (measuring Aβ40, Aβ42, GFAP and NfL) and the Quanterix Simoa p-tau181 measuring p-tau181 of the human tau protein. Assays were performed using the Simoa-HD1 according to the manufacturer’s protocol (Quanterix Corp, Billerica, Massachusetts, USA) (Rissin *et al*). All samples were analysed at the same time using the same batch of reagents. A four-parameter logistic curve fit data reduction method was used to generate a calibration curve. Two control samples of known concentration of the protein of interest (high-control and low-control) were included as quality control. The mean coefficient of variation percentage for p-tau181 was 6.29, for NfL 4.08, for Aβ42 3.06, for Aβ40 2.55 and for GFAP 4.12.

### Statistical analyses

Statistical analyses were performed in R V.4.0.3, R Foundation for Statistical Computing, Vienna, Austria (https://www.R-project.org/). Figures were generated using the R package *ggplot2*. Baseline demographics were compared using analysis of variance and χ^2^ test. The plasma biomarker levels were not normally distributed and were log10 transformed which allowed linear model testing. The Aβ42 and Aβ40 analytes were combined to derive the Aβ42/A40 ratio. To compare baseline plasma biomarkers across groups, we ran an analysis of covariance model to test for the main effect of diagnostic group, accounting for age and sex as covariates using the R package *lme4*. Post-hoc pairwise comparisons were carried out using the Bonferroni correction method for each biomarker tested.

Classification analyses were performed using receiver operating characteristic curve (ROC) analyses to estimate the diagnostic ability of the age adjusted levels of the plasma biomarkers using the *R* package *cutpointr* (https://github.com/thie1e/cutpointr) calculating a cut-off score using the Youden’s index and reporting the area under the curve (AUC), sensitivity (sens) and specificity (spec) for each comparison. The R package *pROC* was used for visualisation of the ROC curves. The classification analyses focused on the ability of the plasma biomarkers to discriminate between MCI+AD and controls, LBD, FTD and PSP.

An analysis of covariance model was used when comparing plasma biomarkers between LBD PET-Aβ-positive and PET-Aβ-negative LBD with age and sex as covariates. Associations between plasma biomarkers and baseline ACE-R were tested in each dementia diagnostic group separately using linear regression models with age and sex as covariates. Longitudinal cognitive decline was measured using a linear mixed effects model applied across the longitudinal cognitive scores to estimate the rate of annual decline (slope) in each participant as previously described in detail.^38^ For each participant, we included ACE-R scores at baseline, 1-year and 2-year follow-up visits where available. The model included the estimation of a random intercept and slope, with time (years) as independent variable and ACE-R scores as dependent variable. The individuals’ estimated rate of annual cognitive decline (slope) was then associated with the plasma biomarkers using a linear regression model, with slope as the dependent variable, the plasma biomarker as the independent variable with age and sex as covariates. Associations between longitudinal cognitive decline and plasma biomarkers were tested in each dementia diagnostic group separately to examine disease specific effects.

## Results

### Participant characteristics

Baseline demographics, cognitive scores and plasma biomarkers levels are shown in table 1. One measurement of GFAP in a control participant was considered an outlier as it exceeded three SD of the mean and therefore this participant was removed from further analyses (GFAP >6000 pg/mL). Age (p=1.6e−11) and sex (p=0.012) were differentially distributed among the groups, in keeping with the known epidemiological characteristics of each neurodegenerative condition. In regression models including diagnosis as a covariate, age was significantly associated with p-tau181 (β=0.007, p=3.24e−05), NfL (β=0.01, p<2e−16) and GFAP (β=0.009, p=4.32e−10) but not with Aβ42/40 (β=0.001, p=0.052). Sex was not associated with significant differences in the levels of the four plasma biomarkers tested. Table 2 summarises the baseline characteristics and biomarker levels of the LBD group stratified per PET Aβ status.

### Plasma biomarkers per diagnostic group

#### P-tau181

**Figure 1:**
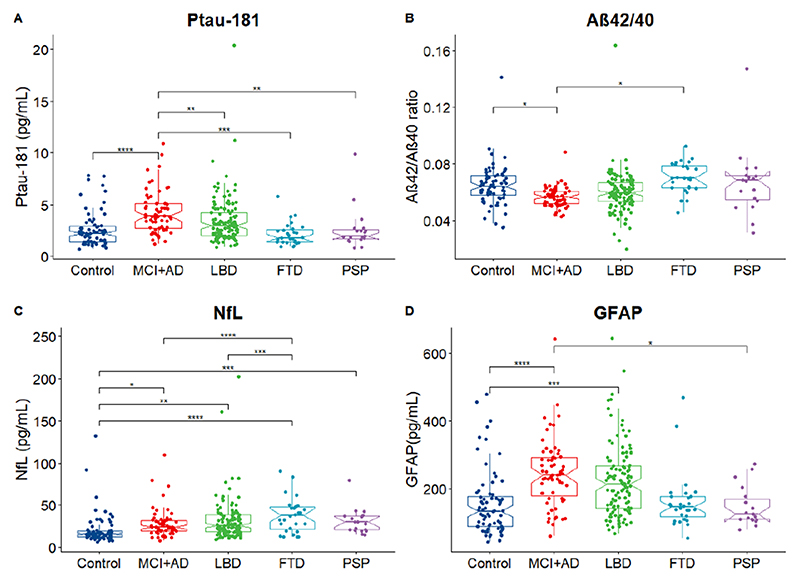
Levels of plasma biomarkers across diagnostic groups. Analysis of covariance revealed a significant effect of diagnosis for all four markers. (A) P-tau181 was elevated in the MCI+AD group when compared with controls, LBD, FTD and PSP. (B) The ratio between Aβ42/40 was lower in MCI+AD group when compared with controls. (C) NfL was elevated across all diagnostic groups when compared with controls and was higher in FTD when compared with LBD and MCI+AD. (D) GFAP was elevated in AD and LBD when compared with controls and in MCI+AD compared with PSP. The Notch graphs display the 95% CIs around the median. Pairwise post-hoc comparisons using the Bonferroni correction are visualised with ****p<00 001, ***p<0001, **p < 0.01, *p < 0.05. Aβ, amyloid beta; AD, Alzheimer’s disease; FTD, frontotemporal dementia; GFAP, glial fibrillar acidic protein; LBD, Lewy body dementia; MCI, mild cognitive impairment; NfL, neurofilament light; PSP, progressive supranuclear palsy; p-tau, phosphorylated tau.

Analysis of covariance revealed significant differences in the levels of p-tau181 between diagnostic groups (F=9.059, p=6.62e−07) after adjusting for age and sex. Post-hoc comparisons using the Bonferroni correction method found higher levels of p-tau181 in the MCI+AD group when compared with all other groups: MCI+AD versus controls (F=5.55, p=6.35e−7), MCI+AD versus LBD (F=3.47, p=6.05e−3), MCI+AD versus FTD (F=4.09, p=5.57e−4) and MCI+AD versus PSP (F=3.56, p=4.36e−3). See figure 1A for schematic representation of results.

#### Aβ42/40

There was a significant effect of diagnosis when comparing the levels of Aβ42/40 among the diagnostic groups (F=3.152, p=0.015). Bonferroni post-hoc comparisons revealed that the Aβ42/40 ratio was lower in the MCI+AD group compared with controls (F=2.84, p=0.0479) and compared with the FTD group (F=2.88, p=0.04) (see figure 1B).

#### NfL

There was a significant effect of diagnosis when comparing levels of NfL among the diagnostic groups (F=14, 9, p=1.38e−10). Bonferroni post-hoc comparisons showed that all dementia groups had higher levels of NfL when compared with controls: controls versus MCI+AD (F=−2.98, p=3.10e−2), versus LBD (F=−3.72, p=2.40e−3), versus FTD (F=−6.90, p=3.31e−10) and versus PSP (F=−4.48, p=1.08e−4). Post-hoc comparisons also showed that patients with FTD had higher NfL levels when compared with MCI+AD (F=4.51, p=9.30e−5) and LBD (F=4.47 p=1.11e−4) (see figure 1C).

#### GFAP

Levels of plasma GFAP were elevated among diagnostic groups when compared with controls (F=0.08, p=1.18e−07). Post-hoc comparisons showed that GFAP was higher in MCI+AD when compared with controls (F=−5.78, p=1.91e−7) and higher in LBD when compared with controls (F=−4.417, p=1.43e−4), while MCI+AD participants also had higher GFAP when compared with PSP (F=3.19, p=1.57e−2) (see figure 1D).

### Classification analyses

#### P-tau181

**Figure 2:**
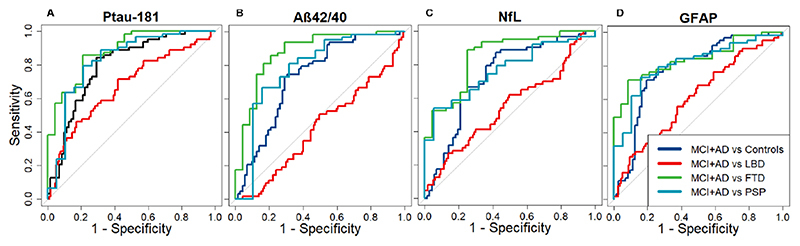
Classification using area under the curve (AUC). (A) P-tau181 could classify MCI+AD from controls with an AUC of 0.80 (sensitivity: 0.84, specificity: 0.70), MCI+AD from LBD with an AUC of 0.67 (sensitivity: 0.58, specificity: 0.71), MCI+AD from FTD with an AUC of 0.88 (sensitivity: 0.85, specificity: 0.79) and MCI+AD from PSP with an AUC of 0.83 (sensitivity: 0.79, specificity: 0.79). (B) The Aβ42/40 ratio could classify MCI+AD from controls with an AUC of 0.72 (sensitivity: 0.75, specificity: 0.69), MCI+AD from LBD with an AUC of 0.42 (sensitivity: 0.50, specificity: 0.51), MCI+AD from FTD with an AUC of 0.88 (sensitivity: 0.86, specificity: 0.79) and MCI+AD from PSP with an AUC of 0.78 (sensitivity: 0.67, specificity: 0.84). (C) NfL could classify MCI+AD from controls with an AUC of 0.73 (sensitivity: 0.87, specificity: 0.58), MCI+AD from LBD with an AUC of 0.55 (sensitivity: 0.27, specificity: 0.86), MCI+AD from FTD with an AUC of 0.85 (sensitivity: 0.89, specificity: 0.75) and MCI+AD from PSP with an AUC of 0.77 (sensitivity: 0.54, specificity: 0.95). (D) GFAP could classify MCI+AD from controls with an AUC of 0.78 (sensitivity: 0.71, specificity: 0.81), MCI+AD from LBD with an AUC of 0.61 (sensitivity: 0.68, specificity: 0.50), MCI+AD from FTD with an AUC of 0.84 (sensitivity: 0.716, specificity: 0.92) and MCI+AD from PSP with an AUC of 0.81(sensitivity: 0.73, specificity: 0.84). Aβ, amyloid beta; AD, Alzheimer’s disease; FTD, frontotemporal dementia; GFAP, glial fibrillar acidic protein; LBD, Lewy body dementia; MCI, mild cognitive impairment with positive PET amyloid scan; NfL, neurofilament light; PSP, progressive supranuclear palsy; p-tau, phosphorylated tau.

An age adjusted cut-off score of 0.43 in the log10 converted levels of p-tau181 could classify MCI+AD from controls with an AUC of 0.80 (sens: 0.84, spec: 0.70). A log10 p-tau181 cut-off score of 0.67 could classify MCI+AD from LBD with an AUC of 0.67 (sens: 0.58, spec: 0.71), a score of 0.65 MCI+AD from FTD with an AUC of 0.88 (sens: 0.85, spec: 0.79) and a score of 0.69 could classify MCI+AD from PSP with AUC of 0.83 (sens: 0.79, spec: 0.79) (see figure 2A)

#### Aβ42/40

An age adjusted cut-off score of 0.46 in the log10 converted Aβ42/40 ratio could classify MCI+AD from controls with an AUC of 0.72 (sens: 0.75, spec: 0.69). Similarly a cut-off score of 0.34 could classify MCI+AD from LBD with an AUC of 0.42 (sens: 0.50, spec: 0.51), a score of 0.64 MCI+AD from FTD with an AUC of 0.88 (sens: 0.86, spec: 0.79) and a score of 0.77 MCI+AD from PSP with an AUC of 0.78 (sens: 0.67, spec: 0.84) (see figure 2B).

#### NfL

An age adjusted cut-off score of 0.39 in the log10 converted levels of NFL could classify MCI+AD from controls with an AUC of 0.73 (sens: 0.87, spec: 0.58). Similarly a cut-off score of 0.41 could classify MCI+AD from LBD with an AUC of 0.55 (sens: 0.27, spec: 0.86), a score of 0.58 MCI+AD from FTD with an AUC of 0.85 (sens: 0.89, spec: 0.75) and a score of 0.81 MCI+AD from PSP with an AUC of 0.77 (sens: 0.54, spec: 0.95) (see figure 2C).

#### GFAP

An age adjusted cut-off score of 0.52 in the log10 converted levels of NfL could classify MCI+AD from controls with an AUC of 0.78 (sens: 0.71, spec: 0.81). Similarly a cut-off score of 0.33 could classify MCI+AD from LBD with an AUC of 0.61 (sens: 0.68, spec: 0.50), a score of 0.71 MCI+AD from FTD with an AUC of 0.84 (sens: 0.71, spec: 0.92) and a score of 0.75 MCI+AD from PSP with an AUC of 0.81 (sens: 0.73, spec: 0.84) (see figure 2D).

### Plasma biomarkers in PET-Aβ-positive LBD

**Figure 3:**
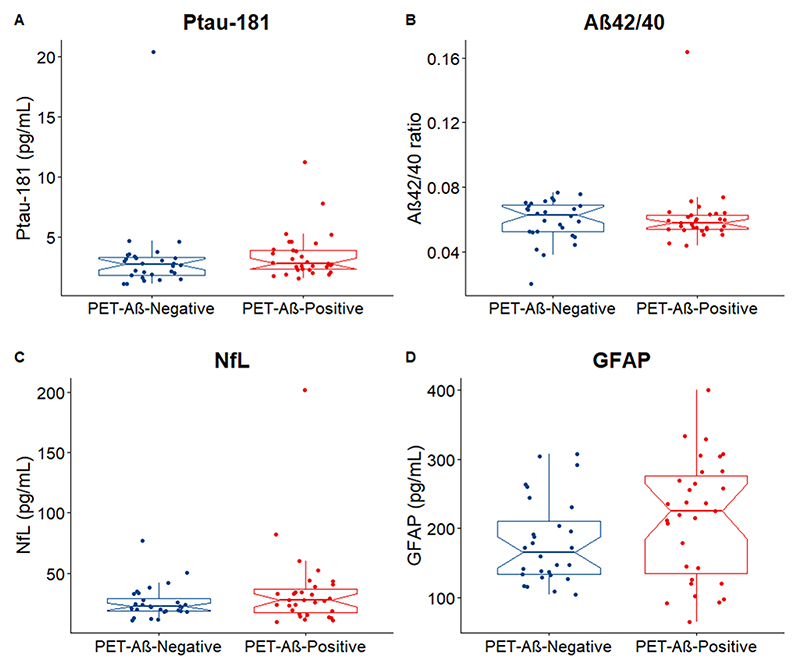
Plasma biomarkers in PET-Aβ-negative versus PET-Aβ-positive LBD cases. No differences were detected when comparing the plasma levels of p-tau181(A), Aβ42/40(B), NfL(C) and GFAP(D) among PET-Aβ-negative(n=30) and PET-Aβ-positive(n=29) LBD cases. The notches display the 95% CI around the median. Aβ, amyloid beta; GFAP, glial fibrillar acidic protein; LBD, Lewy body dementia; NfL, neurofilament light; PET, positron emission tomography; p-tau, phosphorylated tau.

There were no significant differences in the levels of the four plasma biomarkers when comparing patients with PET-Aβ-positive LBD with patients with PET-Aβ-negative (p-tau181 p=0.249, Aβ42/40 p=0.855, NFL p=0.338, GFAP p=0.129; figure 3)

### Association between plasma biomarkers and baseline cognition

#### P-tau181

No associations were found between baseline ACE-R scores and p-tau181 in MCI+AD (p=0.696), LBD (p=0.491) and FTD (p=0.491) after adjusting for the effects of age and sex. P-tau181 was associated with baseline ACE-R scores in PSP (β=−29.17, p=0.026) (see online supplemental figure 1A).

#### Aβ42/40

No associations were found between baseline ACE-R scores and p-tau181 in MCI+AD (p=0.783), LBD (p=0.465), FTD (p=0.544) and PSP (p=0.750) after adjusting for the effects of age and sex (see online supplemental figure 1B).

#### NfL

NfL was associated with baseline ACE-R in the MCI+AD group (β=−26.42, p=0.045). No associations were found between baseline ACE-R scores and NFL in LBD (p=0.07), FTD (p=0.510) and PSP (p=0.662) after adjusting for the effects of age and sex (see online supplemental figure 1C).

#### GFAP

No associations were found between baseline ACE-R scores and GFAP in MCI+AD (p=0.136), LBD (p=0.107), FTD (p=0.471) after adjusting for the effects of age and sex. GFAP was associated with baseline ACE-R scores in PSP (β=−73.64, p=0.019) (see online supplemental figure 1D).

### Association between plasma biomarkers and longitudinal cognitive decline

#### P-tau181

P-tau181 was associated with ACE-R slopes in the MCI+AD group (β=−7.4, p=0.040) after adjusting for the effects of age and sex. No associations were found between ACE-R slopes and p-tau181 in LBD (p=0.382), FTD (p=0.585) and PSP (p=0.205) (see online supplemental figure 2A).

#### Aβ42/40

No associations were found between baseline ACE-R scores and Aβ42/40 in MCI+AD (p=0.348), LBD (p=0.263), FTD (p=0.532) and PSP (p=0.132) after adjusting for the effects of age and gender (see online supplemental figure 2B).

#### NfL

No associations were found between ACE-R slopes and NFL in MCI+AD (p=0.123), LBD (p=0.553), FTD (p=0.308) and PSP (p=0.257) after adjusting for the effects of age and sex (see online supplemental figure 2C).

#### GFAP

GFAP was associated with ACE-R slopes in the MCI+AD group (β=−9.75, p=0.016) after adjusting for the effects of age and sex. No associations were found between ACE-R slopes and GFAP in LBD (p=0.450), FTD (p=0.842) and PSP (p=0.605) (see online supplemental figure 2D).

## Discussion

We show the differential distribution of plasma biomarkers for neurodegeneration in a multicentre clinical cohort comprising patients with PET-Aβ-positive MCI, AD, DLB and Parkinson’s dementia, FTD and PSP. We confirmed that patients in the AD spectrum have high levels of plasma p-tau181 and low Aβ42/40 ratio compared with the other dementias.^1 39^ However, in addition, there are elevated levels of GFAP in both LBD and AD. Patients with FTD and PSP have high NfL but no significant increase in the other biomarkers.

P-tau181 only modestly discriminated between MCI+AD and LBD (AUC 0.67) compared with better discrimination of MCI+AD versus controls (AUC 0.80), FTD (AUC 0.88) and PSP (AUC 0.83). Similarly, Aβ42/40, NfL and GFAP showed good performance in classifying MCI+AD from FTD and PSP but showed limited ability to classify MCI+AD from LBD. The large cohort of well characterised LBD is of particular interest, with biomarker confirmation of the diagnosis through Dopamine Transporter scan (DAT) scan or MIBG in most cases and PET-Aβ scans in half.^23 26 30^ There were no significant differences when comparing the distribution of biomarkers in PET-Aβ-positive LBD compared with PET-Aβ-negative LBD suggesting the limited potential of these plasma biomarkers to identify coexisting AD pathology in LBD, at least with regard to brain amyloid. The p-tau181 and GFAP were associated with baseline cognitive function in PSP, NfL was associated with baseline cognition in the MCI+AD group. Furthermore, the p-tau181 and GFAP were associated with longitudinal cognitive decline in the MCI+AD group suggesting that these may have a potential of prognostic value.

Our findings are in keeping with previous work showing good performance of p-tau181 in discriminating AD from FTD and PSP^4−6^ and we show that the Aβ42/40 ratio, NfL and GFAP perform equally well in such comparisons. Using a large LBD cohort we have however shown that these plasma biomarkers are not as accurate in discriminating between MCI+AD and LBD in contrast to previous work that used smaller LBD cohorts (17 PDD and 6 DLB^6^ and 17 LBD^4^). Moreover our findings of no differences when comparing the levels of plasma biomarkers between PET-Aβ-negative to PET-Aβ-positive LBD are in contrast with previous work suggesting that p-tau181 discriminates LBD cases with and without AD pathology; however that study used CSF Aβ42/40 and PET-tau instead of PET-Aβ.^24^ There can be several factors affecting our results. We observed high variability in both PET-Aβ-positive and PET-Aβ-negative groups which could be related to technical factors such as PET ligand selection and sensitivity. One of our LBD cohorts included PET PIB with positivity defined using the centiloid method while our other LBD cohort used ^18^F-florbetapir with visual rating to define positivity. Considering our sample size it is possible that our study was not powered to detect small effect sizes with high variability. There can also however be underlying neurobiological explanations for not detecting differences between PET-Aβ-negative and PET-Aβ-positive LBD cases. For example, the levels of Aβ pathology in the PET-Aβ-negative cases may have not been sufficient to reach the positivity threshold but may still have affected the levels of p-tau181. Alternatively the presence of α-synuclein pathology may be associated with increase in p-tau independent of the presence of Aβ^40^

The elevated GFAP in AD and LBD is in keeping with previous work of elevated serum and CSF GFAP in these conditions^41 42^ and contribute to the body of evidence pointing towards the involvement of neuroinflammation in neurodegenerative disorders.^19 43 44^ Further research needs to explore the pathophysiology of elevated plasma GFAP and whether it reflects neuroinflammation in specific brain regions or disease stages as, for example, microglial activation in DLB has been linked with early stages of the disease.^43 44^ Interestingly we have not observed elevated levels in of GFAP in FTD and PSP while these have been linked with neuroinflammation using in vivo PET imaging studies.^38 45^ A recent study by Katisko *et al* reported elevated levels of GFAP in serum and whole blood samples of FTD compared with patients with primary psychiatric disorders (PPD) and healthy controls and our analyses in plasma samples were not able to replicate these findings.^46^ It is unclear if the lack of replication is related to our study using plasma samples compared with whole blood or serum used in the study of Katisko *et al* or the age and sample size differences (107 FTD vs 44 PPD serum samples and 10 FTD vs 18 controls whole blood) while the patients with PPD were on average 10 years younger than the patients with FTD.^46^ A study by the Genetic FTD Initiative cohort showed that raised plasma GFAP appears to be unique to progranulin-associated FTD (*GRN* mutation carriers).^47^

Our findings of p-tau181 and GFAP associations with longitudinal decline in ACE-R scores in MCI+AD are in keeping with previous studies showing that these biomarkers have a prognostic value and it will be interesting for future work to test whether they could also have a potential as markers of treatment response.^48 49^ It is difficult to establish the reason as to whether the changes were more pronounced in the MCI+AD group, whether this is a disease-specific finding or associated with limitations in statistical power considering the number of cases available with longitudinal data across the disease groups and the baseline differences. Future studies will also need to replicate our findings with respect to PSP considering the low number of cases available in our cohort.

Our study has several strengths. We tested the clinical utility of the plasma biomarkers in a pooled multicentre clinical cohort of older participants and thus our findings are representative of such clinical populations while we used a cohort with four different types of neurodegenerative disorders. There are however limitations to our study. We had baseline sex and age differences due in part to including neurodegenerative dementias with different epidemiological characteristics which may confound our analyses. We did not have CSF, PET or postmortem confirmation of diagnosis for all our participants and therefore it is likely some of the clinical diagnoses arise from mixed pathologies or alternative pathologies.^22 50 51^ In addition, we had a single biomarker assessment at baseline, so we cannot determine how change in biomarkers over time varies between the different disorders. We had small available number of samples from patients with PSP and FTD while our FTD cohort comprised of mixed pathologies including cases with bvFTD, nfPPA and svPPA with genetic and sporadic cases. Future studies need to validate our findings of elevated plasma markers of neurodegeneration in LBD, FTD and PSP and test the levels of p-tau217 and p-tau231, the association between plasma biomarkers, CSF and neuroimaging.

Collectively our findings suggest that plasma biomarkers in neurodegenerative disorders have a strong potential for diagnosis and monitoring considering their accessibility, convenience, low cost and reproducibility.^52^ We have shown their potential utility in a representative cohort recruited from UK National Health Service memory clinics and showed that each specific neurode-generative disorder has a characteristic pattern that may help clinicians in determining the subtype of diagnosis. Our results highlight the importance of the development of biomarkers for non-AD dementias to complement the current panel of plasma markers in order to better characterise patients with dementia and the different subtypes as this is likely to be of importance if these are used for clinical trials especially with the development of anti-Aβ therapies.^53^

## Supplementary Material

Supplementary Material

## Figures and Tables

**Table 1:** Summary of participant baseline characteristics and plasma biomarker measurements per diagnostic group

	Control (n=73)	MCI+AD (n=63)	LBD (n=117)	FTD (n=28)	PSP (n=19)	P value
Age (years)	70.2 (7.79)	73.9 (7.80)	75.6 (6.81)	64.5 (8.62)	69.0 (5.91)	1.61e−11
Sex (female)	30 (42%)	20 (32%)	23 (20%)	12 (43%)	6 (32%)	0.012
ACE-R	94.5 (4.49)	69.7 (16.5)	66.4 (16.8)	71.0 (15.6)	79.6 (15.3)	<2e−16
P-tau181 (pg/mL)	2.59 (1.65)	4.26 (2.00)	3.49 (2.37)	2.16 (1.09)	2.63 (2.03)	3.24e−05
Aβ42/40	0.0646 (0.0145)	0.0565 (0.00712)	0.0599 (0.0145)	0.0699 (0.011)	0.0671 (0.0237)	0.05207
NfL (pg/mL)	20.9 (18.6)	28.2 (16.8)	32.3 (25.1)	38.2 (20.5)	31.1 (15.0)	<2e−16
GFAP (pg/mL)	154 (96.5)	243 (99.9)	222 (105)	160 (83.9)	146 (57.2)	4.32e−10

Data presented as mean (SD). Comparisons were performed using analysis of covariance. Please note that the statistical tests were performed using the log-transformed values. MCI+AD is the combined group of positron emission tomography Aβ-positive patients with MCI and Alzheimer’s disease (AD), Lewy body dementia is the combined group of dementia with Lewy bodies and patients with Parkinson’s disease dementia.

ACE-R, Addenbrooke’s cognitive examination revised version; FTD, frontotemporal dementia; GFAP, glial fibrillar acidic protein; MCI, mild cognitive impairment; NfL, neurofilament light; pg/ml, picogram/millilitre; PSP, progressive supranuclear palsy; p-tau181, phosphorylated tau at threonine 181; Aβ42/40, the ration between the plasma measurement of amyloid beta (Aβ) 40 and 42.

**Table 2:** Summary of the baseline characteristics and plasma biomarker levels of the Lewy body dementia subgroup that had positron emission tomography-Aβ data available. Data are presented as mean (SD). Comparisons were performed using t-test for age, χ^2^ for sex and a general linear model adjusting for age and sex for ACE-R and the plasma markers

	PET-AβNegative LBD (n=30)	PET-Aβ-Positive LBD (n=29)	P value
Age (years)	73.9 (6.25)	75.2 (6.75)	0.467
Sex (female)	5 (17 %)	6 (21 %)	0.691
ACE-R	74.1 (14.7)	71.3 (11.9)	0.602
P-tau181 (pg/mL)	3.33 (3.49)	3.30 (1.85)	0.476
Aβ42/40	0.0596 (0.013)	0.0613 (0.0208)	0.601
NfL (pg/mL)	25.5 (13.4)	35.8 (35.8)	0.248
GFAP (pg/mL)	179 (62.0)	219 (85.9)	0.181

ACE-R, Addenbrooke’s Cognitive Examination Revised version; Aβ, amyloid beta; GFAP, glial fibrillar acidic protein; NfL, neurofilament light; p-tau, phosphorylated tau.

## Data Availability

Data are available upon reasonable request. Data are available upon reasonable request. Anonymised data may be shared by request to the senior author from a qualified investigator for non-commercial use (data sharing may be subject to restrictions according to consent and data protection legislation).
