# Supplementary material for: Differential levels of plasma biomarkers of neurodegeneration in Lewy body dementia, Alzheimer’s disease, frontotemporal dementia and progressive supranuclear palsy
Source: J Neurol Neurosurg Psychiatry. Author manuscript; Available in PMC 2022 Jun 6. (PMC9148982; doi:10.1136/jnnp-2021-327788)
Supplement: Supplementary Material [file EMS142453-supplement-Supplementary_Material.pdf]

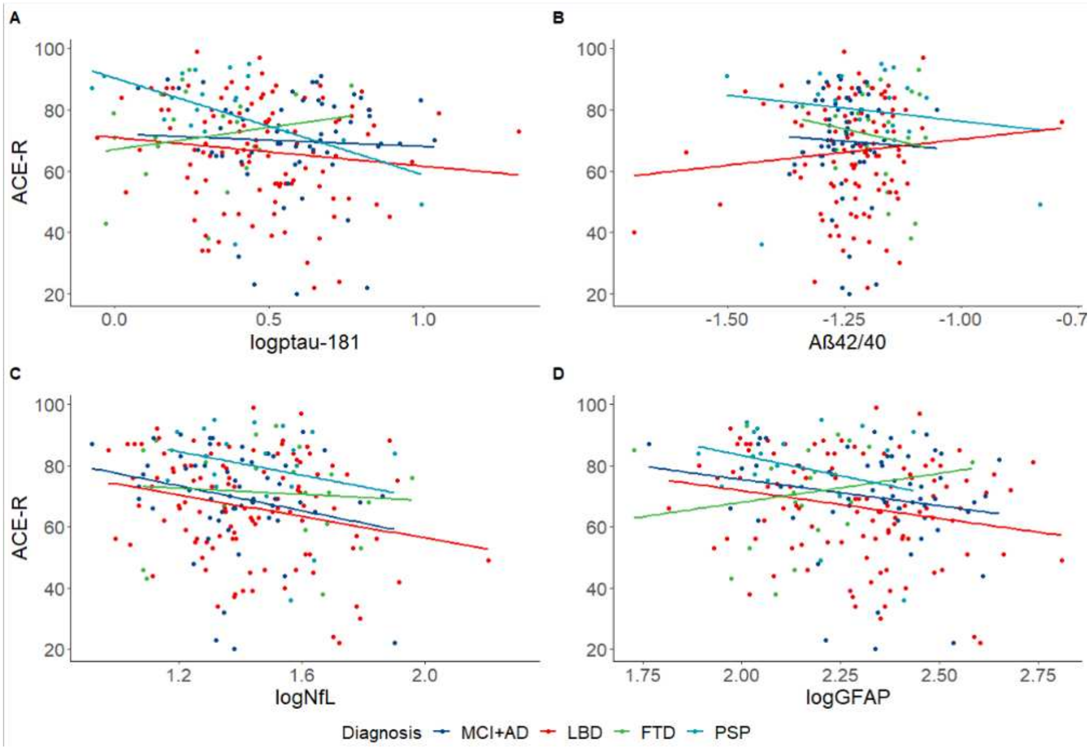

Supplementary Figure 1. Correlations between baseline cognitive function and plasma biomarkers. Baseline cognitive function was measured using the Addenbrooke's cognitive examination-revised version (ACE-R). Associations between ACE-R and the four plasma biomarkers were tested for each diagnostic group separately to examine disease specific effects. Age and sex were used as covariates. Lower ACE-R at baseline was associated with higher levels of Ptau-181 in the PSP group (A; β=-29.17, p=0.026), higher NfL in the MCI+AD group (C; β=-26.42, p=0.045) and higher GFAP in the PSP group (D; (β=-73.64, p=0.019). No associations were detected between ACE-R and Aβ42/40 (B).

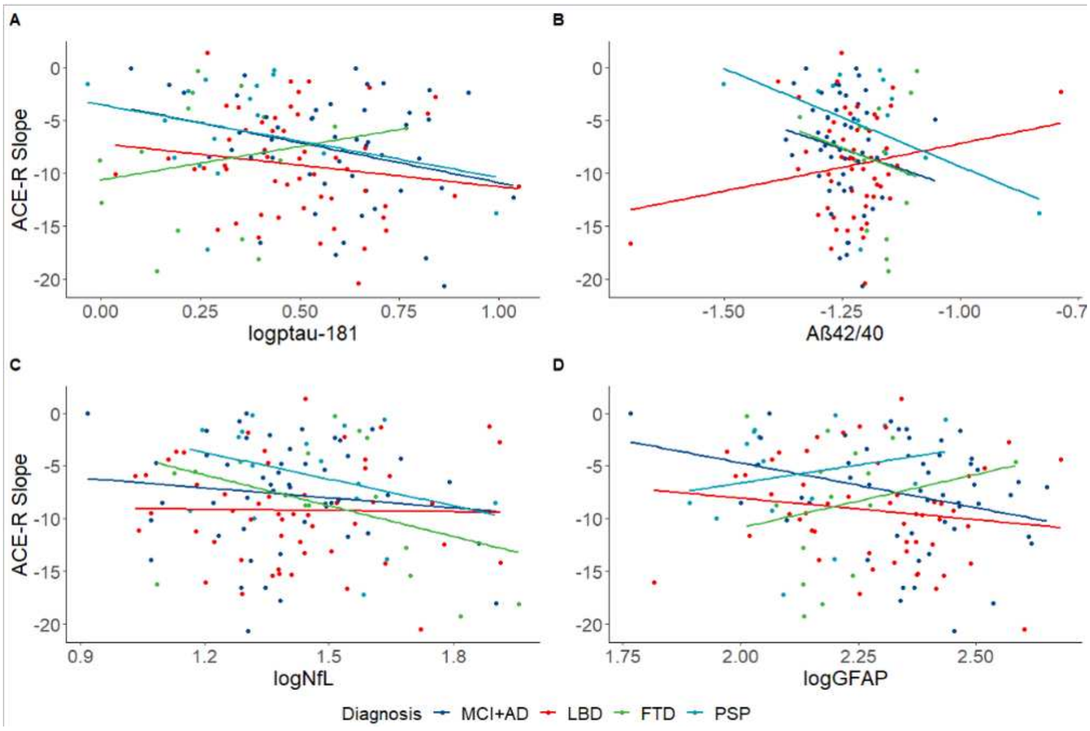

Supplementary Figure 2. Longitudinal cognitive decline and plasma biomarkers. Slopes of longitudinal cognitive decline were extracted from the annual decline in cognitive function measured using the ACE-R. Associations between ACE-R slopes and the four plasma biomarkers were tested for each diagnostic group separately to examine disease specific effects. Age and sex were used as covariates. P-tau181 (A; β=-7.4, p=0.040) and GFAP (D; β=-9.75, p=0.016) were associated with cognitive decline in the MCI+AD group. No significant associations were detected between ACE-R slopes and Aβ42/40 or NfL (B, C).
